# Mitochondrial DNA alterations of peripheral lymphocytes in acute lymphoblastic leukemia patients undergoing total body irradiation therapy

**DOI:** 10.1186/1748-717X-6-133

**Published:** 2011-10-06

**Authors:** Quan Wen, Yide Hu, Fuyun Ji, Guisheng Qian

**Affiliations:** 1 Third Department of Oncology, The second affiliated hospital, Third Military Medical University, Chongqing 400037, China; 2 Institute of Human Respiratory Disease, The second affiliated hospital, Third Military Medical University, Chongqing 400037, China

**Keywords:** mtDNA, 4977-bp Common deletion, Total body irradiation, Real-time-PCR, Acute lymphoblastic leukemia

## Abstract

**Background:**

Mitochondrial DNA (mtDNA) alterations, including mtDNA copy number and mtDNA 4977 bp common deletion (CD), are key indicators of irradiation-induced damage. The relationship between total body irradiation (TBI) treatment and mtDNA alterations in vivo, however, has not been postulated yet. The aim of this study is to analyze mtDNA alterations in irradiated human peripheral lymphocytes from acute lymphoblastic leukemia (ALL) patients as well as to take them as predictors for radiation toxicity.

**Methods:**

Peripheral blood lymphocytes were isolated from 26 ALL patients 24 hours after TBI preconditioning (4.5 and 9 Gy, respectively). Extracted DNA was analyzed by real-time PCR method.

**Results:**

Average 2.31 times mtDNA and 0.53 fold CD levels were observed after 4.5 Gy exposure compared to their basal levels. 9 Gy TBI produced a greater response of both mtDNA and CD levels than 4.5 Gy. Significant inverse correlation was found between mtDNA content and CD level at 4.5 and 9 Gy (*P *= 0.037 and 0.048). Moreover, mtDNA content of lymphocytes without irradiation was found to be correlated to age.

**Conclusions:**

mtDNA and CD content may be considered as predictive factors to radiation toxicity.

## Background

Breakage of cellular DNA following radiation is a dose dependent phenomenon and occurs in both the nuclear and extra-nuclear DNA. Thus, besides nuclear nDNA, mitochondrial DNA (mtDNA) is equally affected as an only extra-nuclear genome [1,2]. Numerous investigations showed that mtDNA can be an easily available target for endogenous reactive oxygen species (ROS) and free radicals caused by ionizing radiation (IR), which resulted in mtDNA copy number alteration and mtDNA damage (such as mutation and depletion) [3,4].

The mechanisms of cellular response to radiation with regard to mtDNA alterations were mainly involved in the following two ways. On one hand, mtDNA has few repair mechanisms and continued mitochondrial function is preserved primarily due to its high copy number. One of possible radio-protective mechanism is that enhanced replication of mtDNA reduces the mutation frequency of total mtDNA and delays the onset of lethal radiation damage to the mitochondria [5,6]. This hypothesis has been recently supported by Zhang et al with exhibiting increased mtDNA copy number in gut and bone marrow of total body irradiated rats [7]. On the other hand, IR usually prompts cell apoptosis by displaying an accumulation of large scale mtDNA deletions, especially the specific 4977 bp deletion, referred to as the "common deletion (CD)" [8]. The site of CD is flanked by two13 bp direct repeats (ACCTCCCTCACCA) at mtDNA nucleotide site 8470 and 13447 respectively, and easy to make deletion for its unique formation mechanism [9]. Studies have shown that CD can be as a sensitive marker of oxidative damage to mtDNA [10-12]. Unfortunately, only few experiments have evaluated the association between CD and IR till now. For example, accumulation of CD has been identified by qualitative PCR method on several irradiated cell lines (such as human skin fibroblasts, glioblastoma and colon carcinoma lines) and primary lymphocytes [13-15]. Furthermore, CD was induced by IR in human hepatoblastoma cell line performing on real-time PCR with nonspecific dsDNA-binding dye SYBR Green. However, their conclusions were largely controversial. The inconsistency may be due, in part, to the use of non-quantitative PCR strategies. Additionally, none of these studies have assayed mtDNA or CD level in peripheral blood lymphocytes (PBLs) after *in vivo *irradiation exposure for lack of appropriate human beings radiation model.

In this study, we performed real-time PCR technique with a specific fluorogenic TaqMan probe conjugated with minor groove binder (MGB) groups, which is more sensitive and appropriate than nonspecific dsDNA-binding dye PCR methods previously used [16]. Besides, we taken the acute lymphoblastic leukemia (ALL) patients undergoging total body irradiation (TBI) precondionting as human beings *in vivo *irradiation model. The advantage of using this model lies in full view of *in vivo *microenvironment, and without need for irradiating healthy individuals. We attempted to address the mtDNA status in irradiated human peripheral blood lymphocytes *in vivo *to elucidate whether alterations in mtDNA can be linked to exposure to total body irradiation.

## Materials and methods

### Study participants

This study comprised peripheral blood (PB) samples from 26 high risked ALL patients undergoing TBI as pre-transplantation treatment in their first complete remission (CR1) at hematology department of our institution. The diagnoses were. according to world healthy organization (WHO) classification and high risk factors were measured on Ribeca's report [17]. The patients age from 19 to 56 years with a mean of 39.4 ± 10.5. Of these, 10 are females and 16 males. Besides, a total of 39 healthy volunteer individuals without IR were included in this study for comparing the difference of basal mtDNA and CD levels between ALL patients and normal donors before IR. The donors age from 18 to 55 years with a mean of 37.2 ± 9.4. 19 are females and 20 males. All tested subjects signed an informed consent to the use of blood samples in accordance with the Declaration of Helsinki and with the approval from our Institutional Review Board. The amount of CD in skeletal muscle under physiological conditions is relatively high (up to 1-2% from total mtDNA content) [18]. Therefore, DNA isolated from skeletal muscle of a 75-year-old male at autopsy was used as positive control in the present study.

### *In vivo *irradiation and peripheral blood lymphocyte isolation

All patients were treated with two 4.5 Gy TBI sessions daily using an Elekta SLi 8 MV linear accelerator (Elekta Co., Stockholm, Sweden) set to deliver a dose rate of 4.5-4.9 cGy/min over two successive days. None of the patients had prior exposure to any cytotoxic treatment for at least 2 weeks before the start of radiotherapy. All patients had 7 ml of PB collected prior to and 24 h following exposure for each radiation treatment. Besides, 39 healthy donors had the same volume of PB collected without ionizing radiation. Preparation of PBLs followed standard methods, using human lymphocyte isolation reagent (TBD Biological Technology Co., Tianjin, China) for separation of mononuclear cells.

### DNA extraction

*DNA *from lymphocytes in vivo and the skeletal muscle was obtained with the TIANamp Genomic DNA Kit (Tiangen Ltd, Beijing, China), and stored at -70°C until further study.

### Analysis of amount of mtDNA and CD by real-time PCR

TaqMan probes with conjugated MGB groups were performed to ensure maximal specificity in real-time PCR reaction. Nuclear DNA content was estimated by measuring the human ß-actin gene. The hypervariable region 2 (HVR2) in the mitochondrial D-Loop was used to represent the total amount of mtDNA since this region is relatively conserved in Han Chinese [19]. The forward primer (ß-actin: 5'-AGGACCCTGGATGTGACAGC-3'; HVR2: 5'-GCTTTCCACACAGACATCATAACAA-3'; CD: 5'-CTTACACTATTCCTCATCACCCAACTAAAAA-3'), reverse primer (ß-actin: 5'-TGGCATTGCCGACAGGAT-3'; HVR2: 5'-GTTTAAGTGCTGTGGCCAGAAG-3'; CD: 5'-GGAGTAGAAACCTGTGAGGAAAGG-3') and TaqMan MGB hybridization probes (ß-actin: 5'-AAAGACACCCACCTTGAT-3'; HVR2: 5'-AATTTCCACCAAACCCC-3; CD: 5'-CATTGGCAGCCTAGCATT-3') were synthesized by GeneCore Bio Technologies Co. Ltd., Shanghai, China. Dose-dependent plasmid-constructed ß-actin, HVR2 and CD standards were used in each run of real-time PCR. Of these, both plasmids containing the CD breakpoint and the HVR2 region were kindly provided by Professor E. Kirches [20]. All TaqMan reactions were carried out in 96-well plates on an ABI 7500 Real-Time PCR instrument (Applied Biosystems, Foster City, CA, USA) using the Real-Time PCR Master Mix kit from Toyobo Co. (Osaka, Japan). Each reaction was carried out in total volume of 25 μl with 50 ng total DNA template, 300 nM each primer, and 100 nM TaqMan-MGB probe. After an initial denaturation step at 94°C for three minutes, 40-45 PCR cycles of 15 s at 94°C, 20 s at 60°C, and 30 s at 72°C were performed. Real-time PCR of all samples and standards were carried out in quadruplicate. The data from a PCR run were rejected if the correlation coefficient was less than 0.98.

### Statistical analysis

All statistical computations were done using the SPSS v15.0 (SPSS, Chicago, IL). Logarithmic transformation of data was essential for further parameter statistical analysis since the original values of the mtDNA and CD copy number in lymphocytes showed a nonnormal distribution. Univariate analysis of variance and Student-Newman-Keuls post hoc tests were used to analyze the difference in mtDNA and CD level with IR exposure. The relative change of mtDNA and CD levels after different dosage exposure were tested by nonparametric Friedman test. The Pearson's correlation test was used to explore association between mtDNA and CD levels. The correlation between mtDNA, CD level and gender, age was analyzed by the nonparametric Spearman's rho correlation test and the Pearson's correlation test individually. *P *values < 0.05 are considered statistically significant. All reported *P *values are two sided.

## Results

### Reliability and reproducibility of the TaqMan-MGB PCR assay

**Figure 1:**
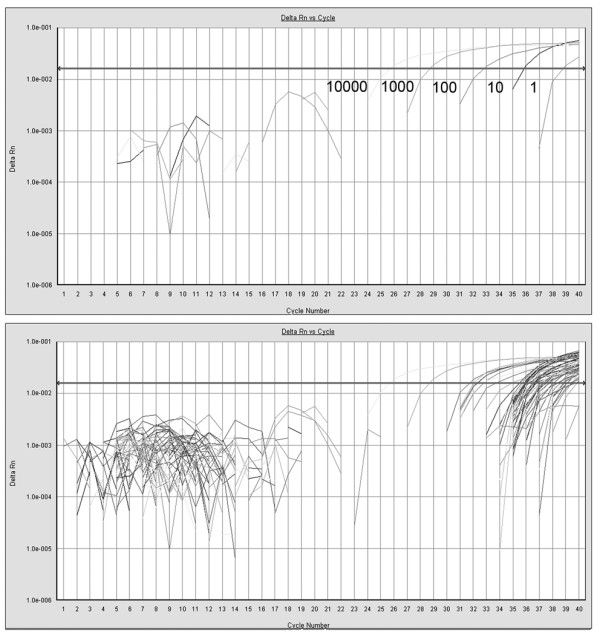
**TaqMan PCR assay for measuring the common mitochondrial deletion in DNA extracted from lymphocytes**. The top panel shows the amplification plot for the standard curve whereas the bottom panel shows the amplification plot for the lymphocyte samples. The level of the common mitochondrial deletion in the lymphocyte samples is within the linear range of the standard curve.

The level of mtDNA and CD from lymphocytes was determined in a set of independent experiments. First, a TaqMan reaction targeting the house keeping gene ß-actin was used to measure the amount of genomic DNA present in cells. A second TaqMan assay was designed to the HVR2 region to quantitate the total amount of mitochondrial DNA. The mtDNA content was normalized to the amount of genomic DNA in a lymphocyte and expressed as a ratio of mtDNA molecules relative to total genomic DNA molecules per cell. A third TaqMan assay targeted the CD breakpoint and measured the abundance of the CD in the samples. The level of CD was normalized using mtDNA amount and was expressed as a ratio to the mitochondrial DNA amounts. In other words, the CD ratio was expressed as a percentage of deleted mtDNA molecules relative to total mtDNA molecules in per genomic DNA molecules. These primer sets have been used extensively for measuring the CD and mtDNA in tissues containing low CD and give reliable results  [20,21]. Figure 1 showed the standard curve for the mtDNA common deletion and the CD amplification plots for the samples examined. It demonstrated that employed TaqMan assay was sensitive enough to detect single molecule of CD and high linearity was found (*y *= 3E^-12e-0.6358^*^x^*) in the range of standard samples. CD levels in most of the samples were detected between Ct 35 and 39. In all samples examined, PCR products were amplified within the linear range of assays (r ^2 ^> 0.98). Positive control DNA from a 75 year old male skeletal muscle contained about 0.729% CD ratio and most of the lymphocytes samples contained from 0.003% to 0.04% CD ratio, consistent with other measurements [18,22]. These results suggest that the TaqMan-MGB PCR approach produces high sensitivity, and could give reliable and corroborating data in our study.

### Basal level of mtDNA content and CD ratio from healthy donors and ALL patients

We first quantified the mtDNA content (median = 197, minimum = 65, maximum = 1124 in ALL; median = 398, minimum = 39, maximum = 1283 in healthy donors) and CD ratio (median = 0.0116%, minimum = 0.0019%, maximum = 0.085% in ALL; median = 0.0193%, minimum = 0.0027%, maximum = 0.121%) per cell in PBLs from ALL patients and healthy donors before irradiation to determine the distribution pattern. Since both variables did not show normal distribution (*P *< 0.01, Kolmogorov-Smirnov test), a logarithm of the mtDNA content and CD ratio was made for normal distributions (see details in additional file 1, Figure S1). Data of mtDNA content and CD ratio after logarithm in the three study groups (0, 4.5 and 9 Gy TBI respectively) were given in Table 1 as mean ± SD, median and range. Mean ± SD values of initial mtDNA and CD level in healthy donors cohort were at 2.507 ± 0.281 and -3.683 ± 0.414. No statistically significant difference was found for logarithm of basal mtDNA and CD level between healthy donors and patients with ALL.

**Table 1:** Logarithm of mtDNA and CD levels in peripheral blood lymphocytes from patients before and after irradiation

Group	Log (mtDNA content)		Log (CD ratio)	
	
	Median (range)	Mean ± SD	Median (range)	Mean ± SD
0 Gy	2.294 (1.811~ 3.051)	2.360 ± 0. 320	-3.934 (-4.730 ~ -3.071)	-3.935 ± 0.459
4.5 Gy	2.566 (1.950 ~ 3.069)	2.526 ± 0. 384	-4.069 (-4.857 ~ -3.063)	-4.148 ± 0. 531
9 Gy	2.715 (1.956 ~ 3.186)	2.711 ± 0. 363	-4.437 (-4.952 ~ -3.255)	-4.233 ± 0.527
*P *^a^		0.038		0.027

Abbreviations: SD, standard devitation; CD, common deletion;

*P *value was demonstrated by univariate analysis of variance.

### Changes of mtDNA content and CD ratio after TBI in patients

Next, we investigated whether the irradiation dose has an effect on mtDNA and CD level with lymphocytes. Significant differences were found between IR status and mtDNA alteration among lymphocytes 24 h after the irradiation (*P *= 0.038 for mtDNA content, 0.027 for CD ratio, Univariate analysis of variance). Furthermore, Student Newman-Keuls post-hoc tests were used to compare the difference among the three groups. mtDNA content was significantly increased in 4.5 and 9 Gy irradiation groups compared with 0 Gy group (mean value of mtDNA content 2.526 and 2.711 compared with 2.360), as well as CD ratio reduced in 4.5 and 9 Gy irradiation groups compared with 0 Gy group (mean value of CD ratio -4.148 and -4.233 compared with -3.935).

### Relative change of mtDNA and CD in lymphocytes from each patient after TBI

**Figure 2:**
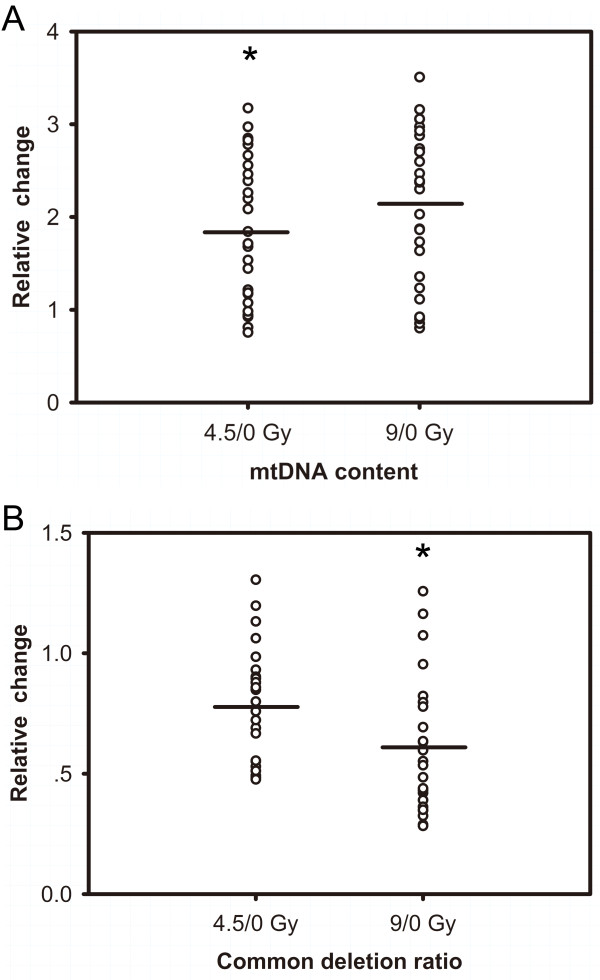
**Relative change of mtDNA content (A) and CD ratio (B) from patients' PBLs (*n *= 26) after different dose of total body irradiation therapy**. Significant difference was observed in relative mtDNA (*P = 0.041) and CD (*P < 0.001) change of every patient when comparing 9 Gy vs. 4.5 Gy exposure. A circle represents mean value of relative change level from each patient undergoing irradiation compared to their basal levels. The lines connect the mean values of relative change level from all cases.

The results above obtained from *in vivo *lymphocytes isolated from patients suggest a correlation of increased mtDNA and decreased CD level with dosage (4.5, 9 Gy) irradiation in cohort study. To better examine the association between mtDNA alterations and IR in individuals, relative changes of mtDNA and CD levels after different dose TBI were compared for each patient. As shown in Figure 2, the increase in mtNDA content was average 1.87 and 2.13 times individually after 4.5 and 9 Gy TBI (*P *< 0.001, Friedman test). Meanwhile, decrease in CD was 0.78 and 0.61 when 4.5, 9 vs. 0 Gy cohorts respectively (*P *< 0.001, Friedman test). Moreover, significant difference was observed in mtDNA copy (*P *= 0.041) and CD ratio (*P *< 0.001) in each patient when comparing 9 Gy vs. 4.5 Gy exposure. Besides, proportions of increased mtDNA content in lymphocytes was found to be 80.8% (21/26) and decreased CD ratio to be 84.6% (22/26) after 4.5 Gy of TBI. Similar trends occurred after 9 Gy exposure, where 84.6% of increased mtDNA content (22/26) and 88.5% of decreased CD ratio (23/26) observed.

### Relation between mtDNA and CD level after irradiation

**Figure 3:**
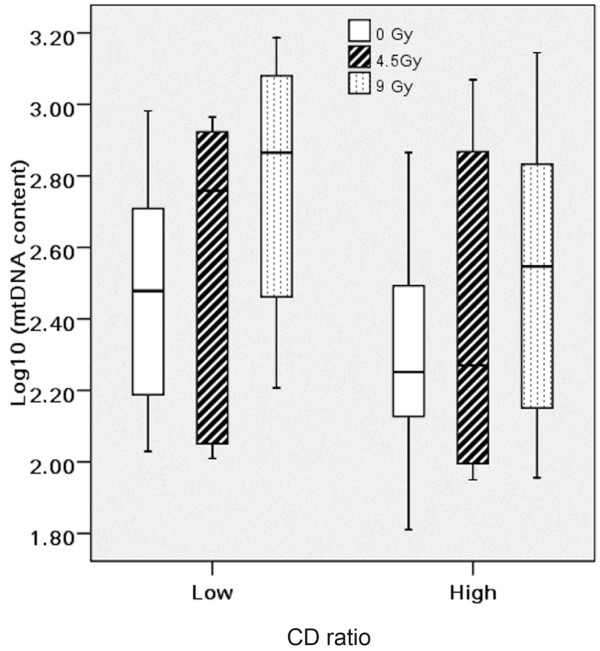
**Box plot shows an association between CD ratio and mtDNA content**. The lines connect the medians, the boxes cover the 25^th ^to 75^th ^percentiles, and the minimal and maximal values are shown by the ends of the bars. Patients with lower amount of CD ratio suffered higher levels of mtDNA.

No relation was found between the level of mtDNA and CD at 0, 4.5 and 9 Gy, when they were analyzed as continuous variables (Pearson test used in all correlations). However, when CD values were segregated in two populations (the lower third against the two upper thirds of the distribution), a modest inverse correlation was found reaching significant level for mtDNA content at different dosage (*P *= 0.037 for 4.5 Gy, 0.048 for 9 Gy, shown in Figure 3). Besides, significant elevated mtDNA content was observed not in high but in low CD population (*P *= 0.021) after 4.5 Gy TBI exposure.

### Effect of age and gender

**Figure 4:**
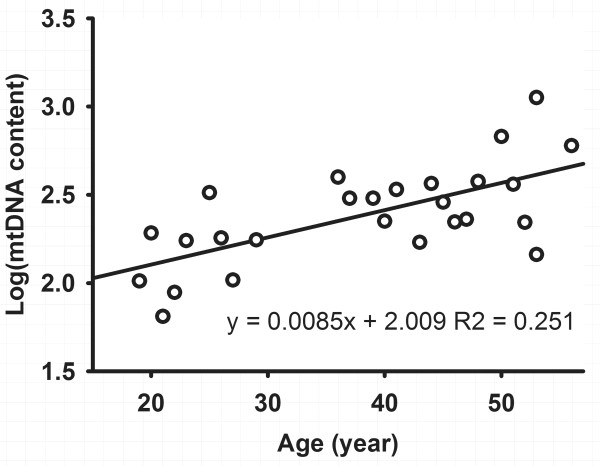
**Regression analysis of the relationship between age and basal mtDNA content from patients' lymphocytes (n = 26)**.

Finally, the correlations between age, gender, mtDNA and CD level were analyzed individually. No relationship was found between mtDNA, CD level and gender. However, a significant positive effect of age was found for basal logarithm mtDNA content in PBLs. A regression analysis allowed quantification of the effect of age on basal mtDNA content (regression coefficient = 0.0085 y-1; r2 = 0.251; *P *= 0.011). The corresponding graphs are presented in Figure 4. These results suggest that older people contained higher mtDNA content in general in the age range of 19-56.

## Discussion

In this paper, we described a sensitive and reliable real-time PCR assay of identifying the mtDNA and common deletion levels. As expected, employed TaqMan-MGB probe was sensitive enough to detect single molecule of CD in our experiment. The sensitivity increased at least 5 fold compared with non specific SYBR Green dye real-time PCR experiment [23]. Besides the improvement of PCR method, we used human tissues and *in vivo *irradiation model, whereas the cell strains and *ex vivo *irradiation model was exclusively used in other studies. As we known, the *ex vivo *cultured cells is unlikely to reflect full view of *in vivo *microenvironment. What is more, a lots of apoptotic cell occurs after IR, which is hardly to isolate from the whole cell population of strain, and will extremely affect the accurate quantification of mtDNA and CD level for cell heterogeneity [23]. In contract, lymphocytes *in vivo *mostly consist of survival cells (> 95%) and could avert the effect of apoptosis [24]. No doubt, it had integrity advantage and is a big step up compared to *ex vivo *model. Based on these evidences above mentioned, we can declare that direct analysis of lymphocytes isolated from human bodies who received TBI would greatly improve specificity and reliability. These technique refinements take us closer to a methodology that is likely to produce reliable and quantitative results.

The role of mtDNA content has been investigated in relation to TBI therapy for the first time in ALL patients. The number of mtDNA copies was elevated in lymphocytes from above 80% of cases after TBI. Besides, mtDNA content of irradiated PBLs elevated consisting with a dose response. This phenomenon has been explained as a compensatory replication of mtDNA to replace damaged mtDNA^7^.

Our statistical analysis showed induced levels of CD after TBI in PBLs, compared to some other reports that IR-induced oxidative stress may cause increase of CD ratio [13,14]. Considering that high deletion level of mtDNA increases the susceptibility of human cell to apoptosis[25], the difference is most likely due to the fact that IR exposure causes lymphocytes differentiate into two major populations immediately: apoptotic population usually containing relative high CD level and thus being sensitive to apoptosis, while surviving population containing relatively low CD level and more resistant to IR. The cell source in other studies is likely mixed with many apoptotic cells, which may resulted in relatively high CD level detection.

Here, we report a statistical inversely association between these two predictive values (mtDNA content and CD ratio) for radiation toxicity. That is to say, lowest values of CD ratio were related to higher values of mtDNA content, at the same radiation dose in our experiment. Cell response to IR is individual, and the amount of initial mtDNA and CD levels depend on each patient. The mechanism behind the relationship remains unclear. One possible reason is that lymphocytes containing lower level of deleted mtDNA have stronger ability to replicate wild mtDNA than cells with high CD level, in order to resist the irradiation induced mitochondrial damage [26]. Besides, abundant mtDNA replication only occurred in low CD population after moderate dosage treatment, which suggests the stronger replication ability of low CD population and a mass of mtDNA copy number production. However, the strong replication ability was not shown in high CD population after modest dosage treatment.

Cellular oxidative stress is thought to play a role in the aging process and may affect mtDNA replication. In the present study, we found that mtDNA copy number is increased with age by lineal regression in our limited cohorts. Similar results has been described that individuals after middle age may be attributed to the enhanced oxidative stress than young adults [27], suggesting age factor should be considered when measuring mtDNA content from both nonirradiated and irradiated lymphocytes.

## Conclusion

This study describes the development of a rapid, sensitive, and practical real-time PCR method to quantify the mtDNA copy number and common deletion in PBL samples. Our results suggest that radiation increased mtDNA content and declined common deletion ratio in peripheral lymphocytes of ALL patients, and an inverse association was observed between both parameters after irradiation, which may be considered as predictive factors to radiation toxicity.

## List of abbreviations

mtDNA: mitochondrial DNA; CD: common deletion; PB: peripheral blood; PBLs: peripheral blood lymphocytes; TBI: total body irradiation; IR: ionizing radiation; HVR2: hypervariable region 2; nDNA: nuclear DNA; ALL: acute lymphoblastic leukemia; MGB: minor groove binder; ROS: reactive oxygen species.

## Competing interests

The authors declare that they have no competing interests.

## Authors' contributions

QW and YH designed the study, FJ provided real-time PCR assay, QW analyzed the data and written the paper, GQ contributed to revising the paper. All authors read and approved the final manuscript.

## Supplementary Material

Additional file 1 **Figure S1**. The histograms show the frequency distribution of logarithm of both mtDNA content (A) and CD ratio (B) from patients (n = 26) after different dose of irradiation. Both population showed normal distributions (*P *= 0.488 and *P *= 0.753 respectively, Kolmogorov-Smirnov test). Click here for file
