# Supplementary figures and images for: Mitochondrial DNA alterations of peripheral lymphocytes in acute lymphoblastic leukemia patients undergoing total body irradiation therapy
Source: Radiat Oncol. 2011 Oct 6;6:133. doi: 10.1186/1748-717X-6-133 (PMC3198693; doi:10.1186/1748-717X-6-133)

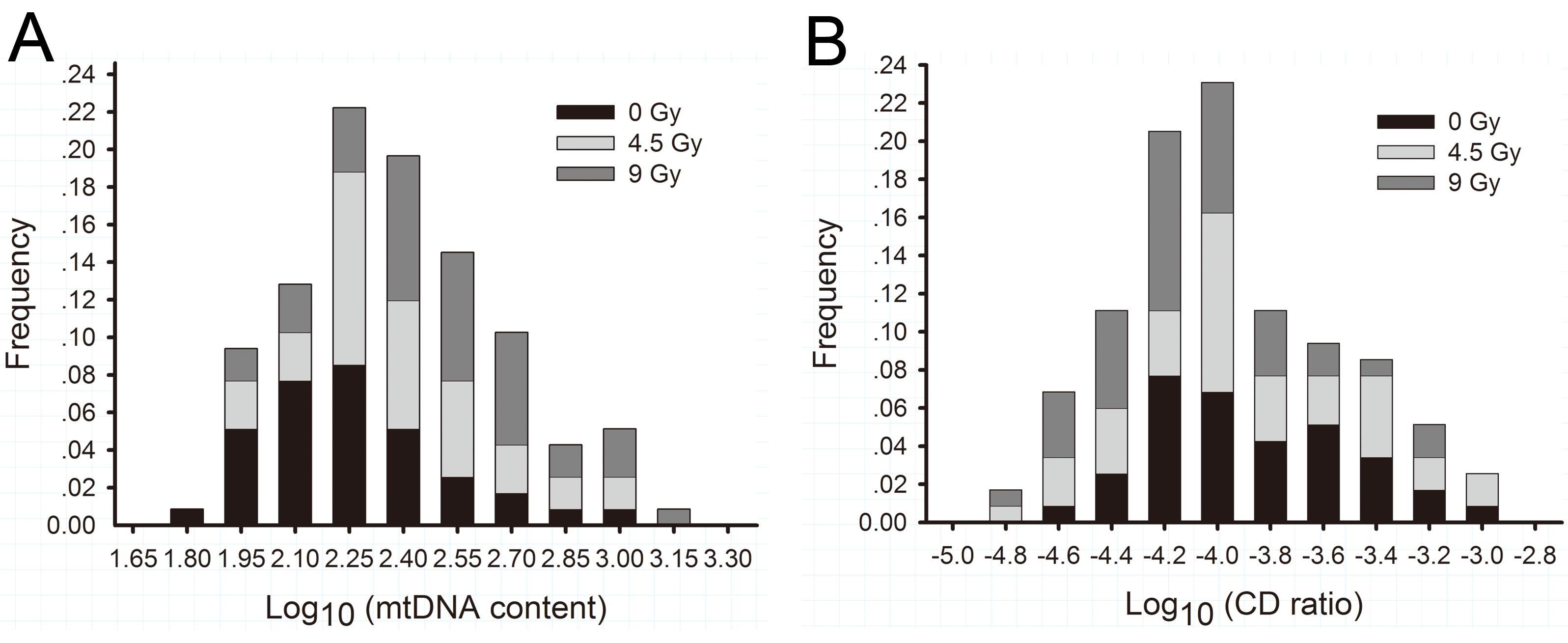

Supplement: Additional file 1 — Figure S1. The histograms show the frequency distribution of logarithm of both mtDNA content (A) and CD ratio (B) from patients (n = 26) after different dose of irradiation. Both population showed normal distributions (P = 0.488 and P = 0.753 respectively, Kolmogorov-Smirnov test). [file 1748-717X-6-133-S1.DOC]
